# Pulse oximetry curves in healthy children living at moderate altitude: a cross-sectional study from the Ecuadorian Andes

**DOI:** 10.1186/s12887-020-02334-z

**Published:** 2020-09-18

**Authors:** Vinicio Andrade, Felipe Andrade, Pablo Riofrio, Fúlvio B. Nedel, Miguel Martin, Natalia Romero-Sandoval

**Affiliations:** 1 School of Medicine, Universidad Internacional del Ecuador, Av. Simón Bolívar and Av. Jorge Fernández. Quito, Quito, Ecuador; 2 Grups de Recerca d’Amèrica i ÀfricaLlatines– GRAAL, Barcelona, Spain; 3 Departamento de Saudé Pública, Universidade Federal de Santa Catarina, Florianópolis, Brasil; 4 Facultad de Medicina, Universidad Autónoma de Barcelona, Barcelona, Spain

**Keywords:** pulse oximetry: reference value, children, altitude

## Abstract

**Background:**

In populations above 3,000 meters above sea level (m.a.s.l.) normal values of oxygen saturation (SpO2) above 90% have been reported. Few studies have been conducted in cities of moderate altitude (between 2,500 and 3,000 m a.s.l). We set out to describe the range of SpO2 values measured with a pulse oximeter in healthy children between 1 month and 12 years of age living in an Ecuadorian Andean city.

**Methods:**

A cross-sectional study was carried out in Quito, Ecuador, located at 2,810 m a.s.l. SpO2 measurement in healthy children of ages ranging from 1 month to 12 years of age residents in the city were recorded by pulse oximetry. Age and gender were recorded, and median and 2.5th and 5th percentile were drawn. Non parametric tests were used to compare differences in SpO2 values by age and gender.

**Results:**

1,378 healthy children were included for the study, 719 (52.2%) males. The median SpO2 for the entire population was 94.5%. No differences were observed between SpO2 median values by age and gender. The 2.5th percentile for global SpO2 measurements was 90%, in children under 5 years of age was 91% and it was 90% in children older than 7.

**Conclusions:**

Our results provide SpO2 values for healthy children from 1 to 12 years old residents in Quito, a city of moderate altitude. The SpO2 percentile curve could contribute as a healthy range for the clinical evaluation of children residing at this altitude.

## Background

Oxygen saturation (SpO2) is an indirect index of oxygen supply-to-demand balance [1, 2]. Pulse oximetry provides information about patient’s oxygenation status and is a reliable, simple, safe, accurate, and relative low cost method to monitor the patient as compared to expensive and labor-intensive methods [3, 4]. Patient’s oxygenation status can show a reduced partial pressure of oxygen and/or decreased oxygen saturation in arterial blood and in this case, it should be called hypoxemia [4]. Hypoxemia in children has been associated with increased mortality and is a frequent complication in cases of pneumonia, bronchiolitis, asthma and other severe diseases such as sepsis [5]. The recognition of hypoxemia among children with pneumonia contributes to diagnosis, is crucial in patient management, and helps in determining prognosis [5–7].

The World Health Organization (WHO) recommends an oxygen saturation threshold value of 90% measured by pulse oximetry, as the cut-off point for oxygen administration in populations living at 2,500 m a.s.l. or less [6]. In clinical practice, the “normal” SpO2 at sea level has been estimated to be between 95% and 100%, however, several authors consider that values of 95% and 96% were abnormal [3]. In altitudes above 3,000 m a.s.l, where oxygen saturation values are lower than at sea level, the 90% cut-off point could be less useful [8].

There are few studies about SpO2 values performed in cities between 2,000 and 3,000 m a.s.l [9–11]. In order to contribute to the best comprehension about SpO2 values in healthy children living at moderate altitude, our study was devised to describe the values range of SpO2 measured with a pulse oximeter in healthy children between 1 month and 12 years of age living at 2,810 m a.s.l.

## Methods

### Subjects and methods

This cross-sectional study was conducted between August 2017 and June 2018. We invited 1,516 children residents in Quito, aged between 1 month and 12 years old, who sought preventive medical attention at three primary health-care centers (Lucha de los Pobres Health-care Center, Cotocollao Health-care Center and Clínica Pichincha), three elementary schools (Escuela Francisco Salazar, Centro del Muchacho Trabajador Cotocollao and Centro del Muchacho Trabajador La Marín) and nine kindergarten municipal schools (Abdón Calderón, Andalucía, Carapungo, Colibrí, Cotocollao, Empleados Municipales, Ipiales, La Carolina and Santa Clara) to participate. Non-probabilistic and convenience sampling were used because age and gender distribution at the schools and health-care centers was unknown. The centers are located at an altitude between 2,740 and 2,901 m a.s.l (average 2,810 m a.s.l). Ambient temperature throughout the study was, on average, 14.4 ^o^C (11.5–20.8 ^o^C) and humidity average was 72.2% (52–81%) as reported by Ecuador’s National Institute of Meteorology and Hydrology[12].

We included children who resided in the city at least 2 months before the study, similar criteria used in other study [13], and children younger than 2 months must have been born and remain living in the city until they were examined. Exclusion criteria included a registered axillary temperature > 37.5 C° at the time of evaluation, history of respiratory symptoms in the two weeks prior to evaluation, any abnormal cardio-respiratory signs during physical examination, history of chronic cardio-respiratory disease, history of neonatal respiratory disease, history of blood component transfusion in the six months prior to evaluation, and the presence of malnutrition, defined as a Z-score less than − 2SD for either height for age or weight for height [9, 10].

Children were enrolled in the study after written informed consent obtained from their parents. The study was approved by the Universidad Internacional del Ecuador Ethics Committee, registered code CEU-005-16 and by the health committee at each center participating in the study. Information on the study was provided to the directive councils and medical teams at each institution.

Fifteen students from fourth year of a school of medicine were rigorously trained in anthropometric measurements and pulse oximetry assessment by the standardization method of the Central America and Panama Institute of Nutrition (INCAP) [14]. To manage measurement bias, the students’ measurements were compared against a pediatrician’s reference pattern, establishing a maximum margin of error of 0.2 kg for weight and 0.5 cm for length and height [14].

### Variable definition

Weight and height were measured using high fidelity equipment (Health-o-Meter 498KL and 593KL, USA), regulated and previously calibrated by the Ecuadorian Institute of Normalization. Respiratory frequency was obtained through observation in calm and alert children, visually counting thoracic and abdominal movements over one minute. Temperature was assessed with a flexible digital thermometer (Omron MC-343F, Mexico), placed in children’s armpit until a reading signal was obtained. Heart rate and SpO2 were evaluated using automatically calibrated non-invasive pulse oximeters (Huntleigh MP1R Smartsigns® MiniPulse Huntleigh Healthcare Ltd, Cardiff, United Kingdom). The pulse oximeter used measured functional oxygen saturation with a precision range of ± 2%. Pulse oximetry was assessed in calm and alert children. Wrap-around style and fold-over-style probes were used, depending on the subject’s age, and placed either on right hand’s index finger or the big toe for infants. Nail polish remover was provided for subjects who had nail polish present at the time of the test. The SpO2 measurements were considered adequate when a plethysmographic waveforms of perfusion levelled-off on its high end and remained on the output screen for at least 2 minutes. Then, SpO2 measurements and pulse rate, were recorded every 10 seconds for a total of three measurements, and the average was used to determine SpO2 for each study subject [6, 15–17].

### Statistical Analysis

Descriptive statistical tests were run for clinical measurements, and 2.5th percentile, 5th percentile, 25th percentile (Q1) and 75th percentile (Q3) for SpO2 distribution. The Kruskal-Wallis test was used to compare differences in SpO2 medians by age groups, Mann-Whitney U test was used to compare medians between males and females. Statistical significance was accepted with *p* < 0.05. Smooth lines were designed for percentiles 2.5th and 5th for SpO2 using the Spline method (*smooth.spline* function in R, with a 7 degree freedom range). All data was registered in the digital survey platform Survey Monkey®, and analyses were performed using SPSS®, version 24. Graphics were designed using R version 4.3.

## Results

1,516 children were invited to participate. A total of 1,378 (90.9%) subjects were included for the study, of which 719 (52.2%) were male. 138 children were excluded of which 7 were for not being residents of Quito, 56 had fever, 141 presented any cardiorespiratory symptoms, 31 presented stunting and 15 acute malnutrition. 13 had history of chronic cardiorespiratory disease, 3 had history of neonatal respiratory disease, and 1 had blood transfusion prior. 55 (39.8%) had only one exclusion criteria and 8 (5.8%) had 4. Measurements characteristics of the included group are listed in Table 1.

**Table 1:** Clinical measurements characteristics by age group (mean and standard deviation)

Age (years)	n (percentage of total)	HR beats/min mean (SD)	RR breaths/min mean (SD)	BMI Kg/m^2^ mean (SD)	Height m mean (SD)	Temp ^o^C mean (SD)
< 1	167 (12.1)	132.8(14.3)	43.6 (9.2)	16.6(1.8)	0.64(0.1)	36.8(0.2)
1	58 (4.2)	125.4(12.0)	33.8(5.4)	16.4(1.4)	0.78(0.1)	36.7(0.4)
2	149 (10.8)	113.4(10.9)	30.1(4.4)	16.27 (1.4)	0.87(0.0)	36.6(0.4)
3	154 (11.2)	105.9(11.2)	27.1(3.6)	16.0(1.3)	0.95(0.1)	36.6(0.4)
4	155 (11.2)	100.6 (11.2)	26.0 (3.3)	16.0(1.4)	1.00(0.0)	36.6(0.4)
5	111 (8.1)	98.4(11.9)	25.9 (3.1)	16.0 (1.3)	1.06(0.1)	36.6(0.4)
6	74 (5.4)	88.8(12.6)	24.5(3.8)	16.1(2.4)	1.13(0.1)	36.7(0.4)
7	101 (7.3)	91.7 (12.4)	26.1(3.5)	16.3(1.9)	1.19(0.6)	36.6(0.4)
8	93. (6.7)	88.0 (11.1)	25.9 (3.9)	17.0 (2.3)	1.24(0.1)	36.7(0.4)
9	96 (7.0)	87.9(12.6)	25.2(4.3)	17.0 (1.9)	1.28(0.6)	36.6(0.4)
10	85 (6.2)	84.0(11.4)	24.8(3.9)	17.5 (2.1)	1.33(0.1)	36.5(0.4)
11	100 (7.3)	82.2(11.1)	24.1(3.9)	18.1(2.4)	1.38(0.1)	36.6(0.4)
12	35 (2.5)	83.0 (11.3)	24.0 (2.7)	18.1(1.9)	1.41(0.1)	36.5(0.4)

Heart Rate (HR) defined as beats per minute. Respiratory Rate (RR) defined as breaths per minute. Body Mass Index (BMI). Height measured in meters (m) and Body temperature measured in Celsius degrees (°C).

The overall SpO2 lowest and highest values were 87% and 99%. Median, 2.5th, 5th, 25th (Q1) and 75th percentile (Q3) for SpO2 by age are listed in Table 2.

**Table 2:** Distribution of oxygen saturation measured by pulse oximetry by age

Age (years)	Median	2.5th Percentile	5th Percentile	25th Percentile (Q1)	75th Percentile (Q3)
< 1	95.1	90.0	91.3	93.7	96.7
1	95.3	91.9	92.7	94.3	96.0
2	95.0	90.7	91.8	93.7	96.0
3	95.0	91.8	92.3	94.0	96.0
4	95.0	90.6	91.6	94.0	95.7
5	94.3	91.3	91.7	93.3	95.3
6	94.3	90.4	91.2	93.3	95.7
7	94.3	90.2	91.6	95.3	95.7
8	94.3	90.2	91.6	93.3	95.3
9	94.3	89.2	91.6	93.0	95.3
10	94.3	90.7	91.3	93.3	95.3
11	94.0	91.0	96.7	93.0	95.0
12	93.8	90.7	91.8	92.8	94.7
Global	94.7	90.7	91.7	93.4	96.7

SpO2 at 12 years of age was the lowest median value (94%), and the highest median value was observed in children aged 1 year (95%); no significant differences in SpO2 median values were found between age (Kruskal-Wallis Chi square test = 7.94, df = 11, *p* = 0.72).

**Fig. 1:**
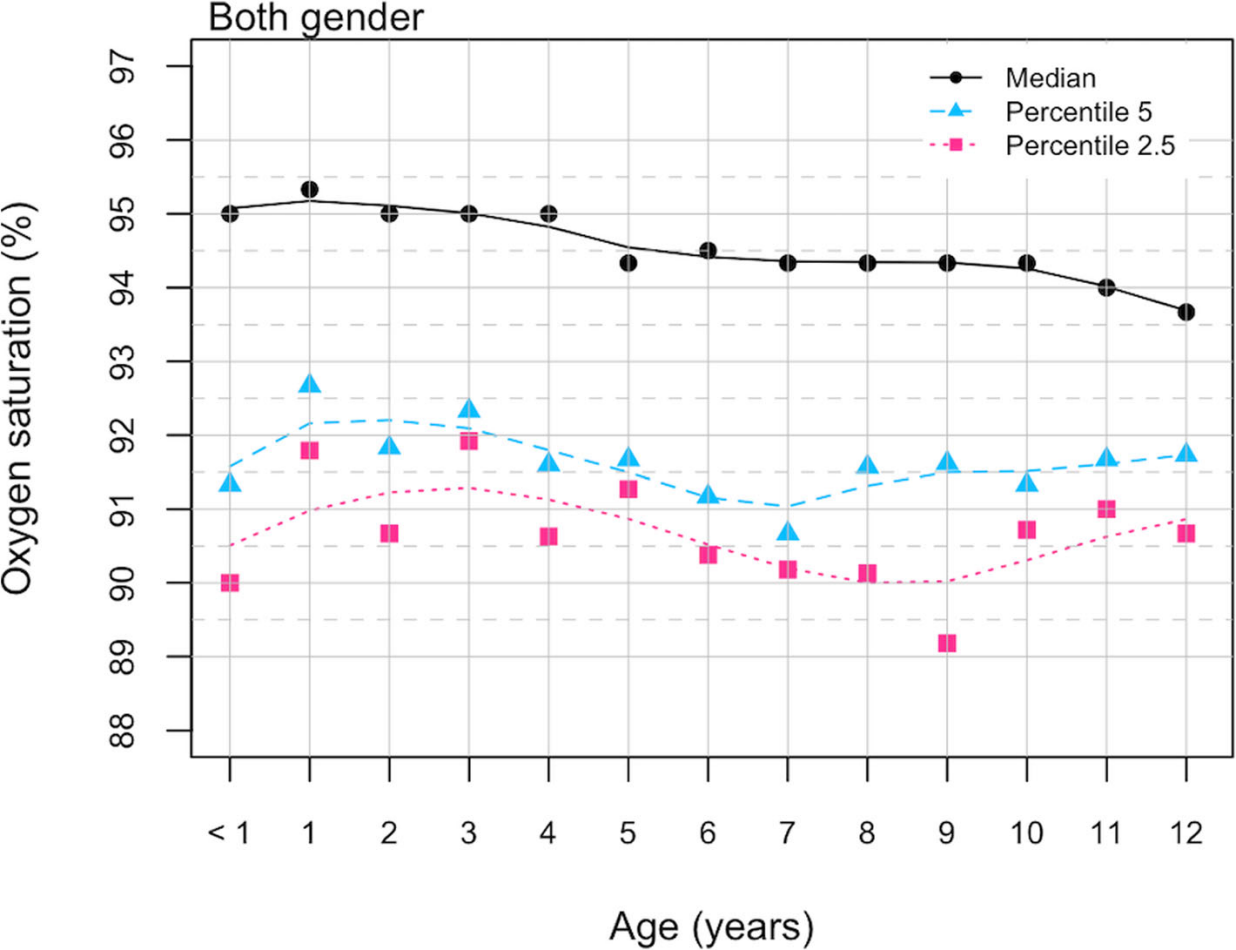
SpO2 percentile values by age. Children residents in Quito, august 2017-june 2018

Figure 1 shows the smooth percentile lines for SpO2 corresponding to percentiles 5th and 2.5th in all participants by age. It is noteworthy that in children between the ages of 7 and 9 the SpO2 value for percentile 2.5th was between 89% and 90%, while in other age groups the values recorded were between 90 and 91%.

**Fig. 2:**
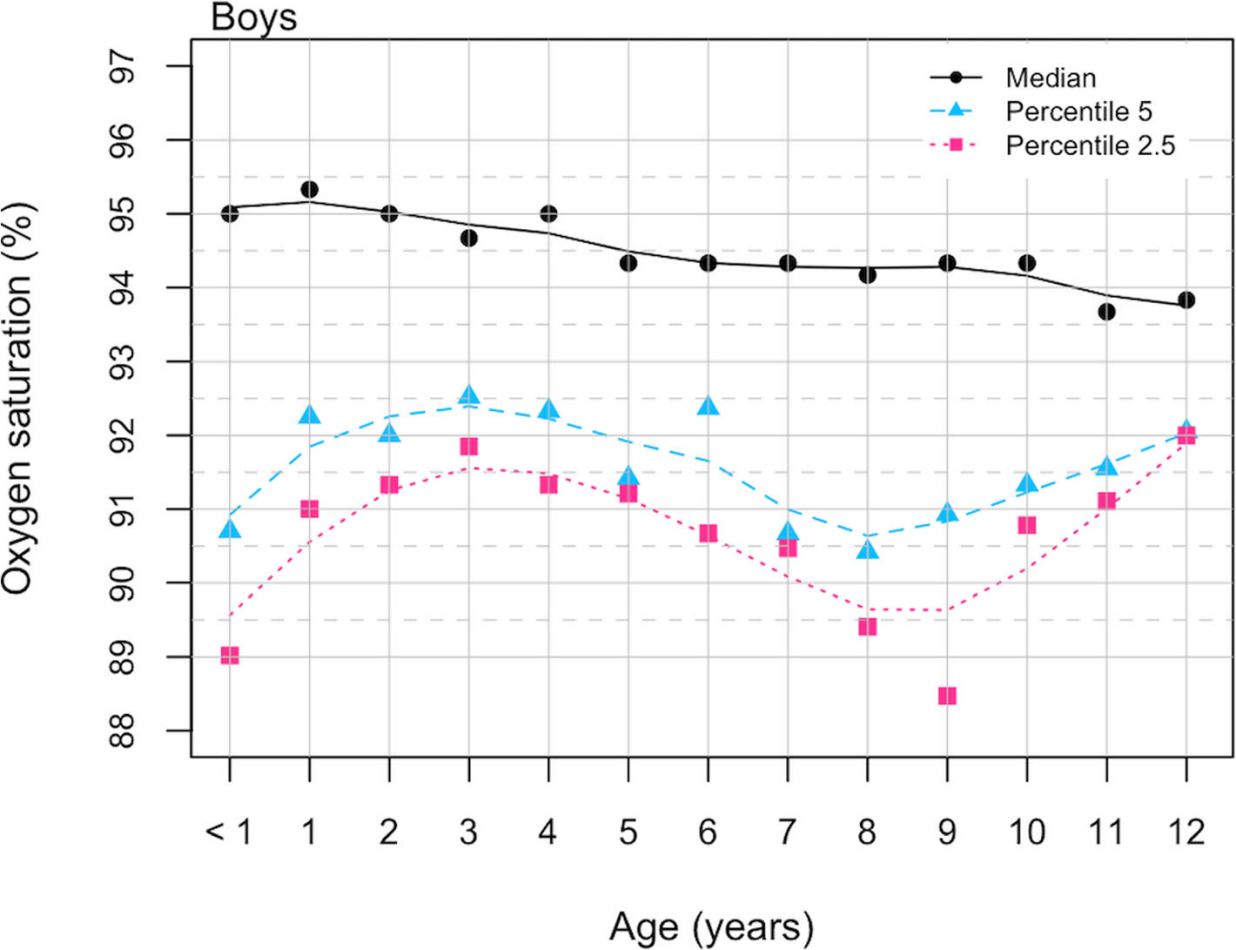
SpO2 percentile values for age in boys residents in Quito, august 2017-june 2018

**Fig. 3:**
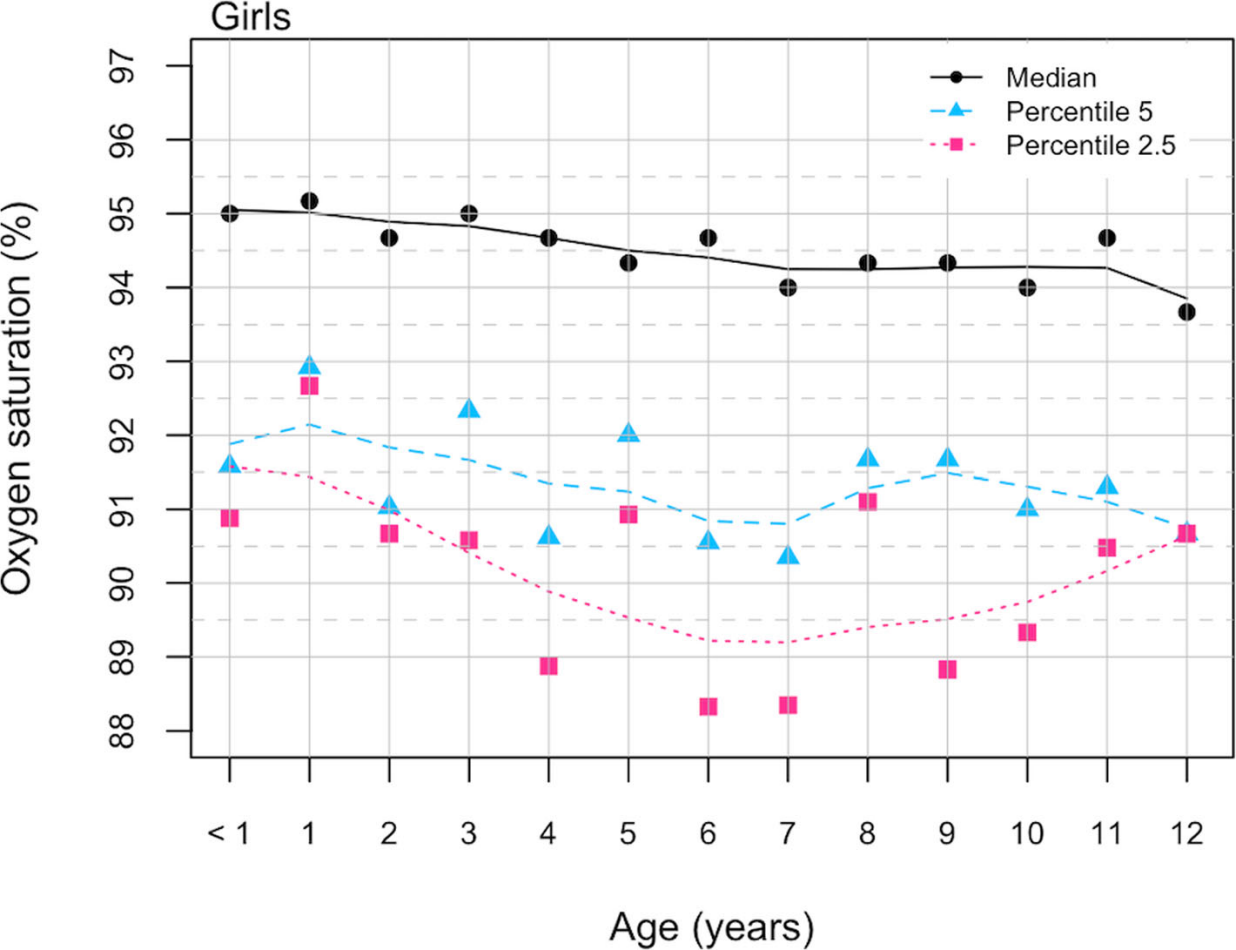
SpO2 percentile values for age in girls residents in Quito, august 2017-june 2018

Figure 2 represents SpO2 percentile lines for male population, value percentile 2.5th for SpO2 was between 89% and 90% for children younger than 1 year and of 8 and 9 years of age, respectively. Figure 3 represents the same data for females. Values 2.5th percentile for SpO2 were between 89% and 90% from 4 to 11 years of age. No differences were observed by gender (Mann-Whitney U test, z=-1,095 *p* = 0.273).

## Discussion

The use of pulse oximetry is advised in order to increase detection of hypoxemia, considering normal SpO2 range values ​​at higher altitudes [5, 8]. Some studies that reported SpO2 measurements by average have showed SpO2 value is close to 99% at sea level and appears to decrease to 97% after 1500 to 1600 m a.s.l [15, 17, 18]. Furthermore, at 3000 m a.s.l, mean SpO2 values ​​of 89.6% [19] or even 85.7% have been reported [20]. Lozano et al. at Bogotá-Colombia (2600 m a.s.l) have reported mean SpO2 value of 93.3% (SD 2.05%) [10] and Nicholas et al. at Colorado-USA (2800 m a.s.l) of 91.7% (SD 2.1%) [9]. Because SpO2 levels are not normally distributed, it seems appropriate to report the data in median values ​​and percentile values [8]. We present a curve with the medians and 5th and 2.5th percentiles of SpO2 of children from 1 month to 12 years permanent residents in the city of Quito, at 2810 m a.s.l. We did not find significant differences by children age or sex.

The median for global SpO2 measurements was 94.67%, 5th percentile was 91.67%, and 2.5th percentile was 90.67%. Rojas-Camayo et al. in children aged 1 to 5 years, report at 2880 m a.s.l, SpO2 median value of 95%, 2.5th percentile was 91% [21]. In a recent study, Tüshaus et al. obtained an altitude-adaptive SpO2 computer model and proposed a model derived altitude-adaptive abnormal SpO2 threshold for an abnormal range that could indicate hypoxemia, in healthy children living permanently at altitudes up to 4000 m a.s.l [11]. They used the physiological model of the oxygen cascade and incorporated the technical tolerances that accounted for the accuracy of pulse oximeters (+ -2%). With this model, the median at 2800 m a.s.l was 93% with lower healthy range of 88%, and abnormal SpO2 threshold of 86%. To evaluate the model, the authors compared it with an empirical dataset of 297 children residing between 2000 and 3900 m a.s.l. In that empirical study, they report that at an altitude of 2800 m a.s.l median SpO2 was 95.2% and 2.5th percentile was 90%[11]. Our median and 2.5th percentile values presented similar results to those of Rojas-Camayo and the empirical study of Tüshaus et al. According to the physiological model of Tüshaus et al., the median value is similar, but the 2.5th percentile is lower. It is possible that this difference is due to the fact that the altitude-adaptive model is physiological and takes into account the pulse oximeters accuracy.

Pulse oximetry is a non-invasive and relative low-cost assessment method able to reduce child mortality by accurately diagnosing hypoxemia, increasing the possibilities of early and effective treatment [21]. Unreal SpO2 values could increase hospital admissions and hospital stays with subsequent iatrogenic risks and misuse of resources [21, 22]. Without pulse oximetry, the management of pediatric patients depends on precise identification of the clinical signs of hypoxemia, which are not always easy to assess in all patients. Clinical signs alone are unreliable for the detection of hypoxaemia [23].

Pulse oximetry identifies between 20 and 30% more cases than clinical signs alone [21, 22, 24, 25]. However, the determination of a threshold to identify hypoxemia is difficult, especially in high altitude populations [26]. Some studies have previously used average SpO2 values ​​-2 SD to define hypoxemia, however others have used the 2.5th percentile measurement as a cut-off point to decide to use oxygen [8]. Subhi et al., developed a statistical model of SpO2 distribution from sea level to 4000 m a.s.l. using meta regression based on 14 observational studies of healthy children. At an altitude of 2500 m a.s.l the 2.5th percentile was 90% and at 2800 m a.s.l was 88%. It suggests that for altitudes greater than 2500 m a.s.l the SpO2 threshold for identifying children requiring oxygen is 85% although it is not clearly defined how the threshold was chosen [8]. The threshold obtained with the Tüshaus altitude-adaptive model at 2800 m.a.s.l was 86% [11]. It is possible, that our results are related to “normality” in “children at community settings without respiratory symptoms and not fever living at moderate altitude”, however, the 2.5th percentile values ​​could be conservative to be considered as a threshold to define hypoxemia or the need to administer supplemental oxygen. It is very important to consider the accuracy range of the pulse oximeter used (+-2%) [11].

Physicians should consider the patient’s clinical condition to make the decision to use oxygen therapy, especially in places where we have limited availability of resources. It would be important to evaluate SpO2 in children with low respiratory disease at moderate altitude to propose cut-off points for supplemental oxygen administration.

There are some limitations to the study. (1) Measurements were carried out in children between 1 month and 12 years of age living in Quito, so the results obtained cannot apply to patients who have not adapted to altitude. (2) All children in the sample had their medical records and physical examination taken but did not undergo laboratory testing for parameters such as serum hemoglobin, arterial blood gas testing or chest X-rays to discard other pathologies not found on clinical evaluation. (3) Although we carry out a standardization of personnel in the use of the pulse oximeter, we cannot totally exclude possibilities of error in the measurement of SpO2 such as incorrect positioning of the probe or insufficient perfusion. (4) In this study we did not set out to compare the SpO2 results between the group that met the inclusion criteria with the excluded group; However, this comparison could provide additional information.

## Conclusions

Our results provide SpO2 values for healthy resident children from 1 to 12 years old at 2810 m. a.s.l., which could be considered as healthy ranges. SpO2 percentile curve could contribute as a reference range for the clinical evaluation of resident’s children at this altitude.

## Data Availability

Non-identified individual participant data (including data dictionaries) will be made available, in addition to study protocols, the statistical analysis plan, and the informed consent form. The data that support the findings of this study are available in DSpace in the following identifier https://repositorio.uide.edu.ec/handle/37000/4228. The data will be made available upon publication to researchers who provide a methodologically sound proposal for use in achieving the goals of the approved proposal. Proposals should be submitted to vinanmay@gmail.com.

## Abbreviations

SpO2: Oxygen Saturation
WHO: World Health Organization
m a.s.l: Meters above sea level
UIDE: Universidad Internacional del Ecuador
